# Denosumab Discontinuation and the Rebound Phenomenon: A Narrative Review

**DOI:** 10.3390/jcm10010152

**Published:** 2021-01-04

**Authors:** Athanasios D. Anastasilakis, Polyzois Makras, Maria P. Yavropoulou, Gaia Tabacco, Anda Mihaela Naciu, Andrea Palermo

**Affiliations:** 1 Department of Endocrinology, 424 General Military Hospital, 564 29 Thessaloniki, Greece; 2 Department of Endocrinology and Diabetes and Department of Medical Research, 251 Hellenic Air Force & VA General Hospital, 115 25 Athens, Greece; pmakras@gmail.com; 3 Endocrinology Unit, 1st Department of Propaedeutic and Internal Medicine, School of Medicine, National and Kapodistrian University of Athens, 115 27 Athens, Greece; maria.yavropoulou.my@gmail.com; 4 Unit of Endocrinology and Diabetes, Campus Bio-Medico University, 00128 Rome, Italy; g.tabacco@unicampus.it (G.T.); a.naciu@unicampus.it (A.M.N.); a.palermo@unicampus.it (A.P.)

**Keywords:** denosumab, discontinuation, fracture, rebound, osteoporosis, turnover

## Abstract

Denosumab is a potent antiresorptive agent that substantially increases bone mineral density and reduces fracture rates at all skeletal sites for as long as it is administered. However, its favorable skeletal effects reverse quickly upon its discontinuation, because of a vast increase of osteoclast number and activity, which leads to a subsequent profound increase of bone turnover above pre-treatment values, a phenomenon commonly described as “rebound phenomenon”. More importantly, most patients experience rapid, profound bone loss due to this burst of bone resorption that may lead in a minority of these patients to occurrence of fractures, especially multiple vertebral fractures. Therefore, subsequent antiresorptive treatment is mandatory, although the optimal regimen is yet to be clarified. In the present review, we outline what is currently known regarding the negative effects of denosumab discontinuation on different aspects of bone status, the factors that may affect them, and strategies to prevent them.

## 1. Introduction

Osteoporosis is a chronic disease, requiring long-term management. Although osteoanabolic agents, such as parathyroid hormone (PTH) analogs and romosozumab, achieve impressive increases of bone mineral density (BMD), they can be administered only for a relatively short period of 1–2 years. Therefore, antiresorptive agents still remain the cornerstone of osteoporosis treatment [1,2]. Among them, a commonly preferred option is denosumab (Dmab), which has been reported to gradually increase BMD and persistently reduce fracture rates at all skeletal sites for as long as it is administered [3]. Additionally, its relatively easy and convenient way of administration along with a fair safety profile render Dmab an excellent choice in the long-term management of osteoporotic patients. Unfortunately, its favorable skeletal effects reverse quickly following its cessation [4].

In this review, we summarize the most recent evidence regarding the negative effects of Dmab discontinuation on different aspects of bone status, the factors that may affect them, and strategies to prevent them.

## 2. Methods

We searched for articles published in PubMed and Cochrane Library from inception up to November 2020, to identify published original articles concerning Dmab discontinuation. In particular, we searched for articles that directly or indirectly investigated the consequences of Dmab discontinuation on bone health. The term “denosumab discontinuation” was matched with the following terms: C-terminal telopeptide of type I collagen (CTX), N-terminal propeptide of type I procollagen (P1NP), bone turnover markers (BTM), BMD, fracture, osteoblast and osteoclast, osteoporosis, rebound phenomenon, bisphosphonate, alendronate, risedronate, ibandronate, zoledronate.

We searched for articles published in English and to minimize differences, studies were included if they met the following criteria: (1) those that were cohort studies, case-control studies, or cross-sectional studies, case reports or case series; (2) the exposure of interest was Dmab use/discontinuation, the outcomes were BMD loss, BTM changes, fractures, and osteoporosis. Exclusion criteria included non-primary research, review articles, lack of a primary outcome related to the relationship between Dmab discontinuation and bone health, or non-English language publications. Two investigators (G.T. and A.M.N.) independently searched papers, screened titles and abstracts of the retrieved articles, reviewed the full-texts, and selected articles for their inclusion.

## 3. Mechanism of Dmab Action—Effect of Discontinuation on Bone Metabolism

**Figure 1:**
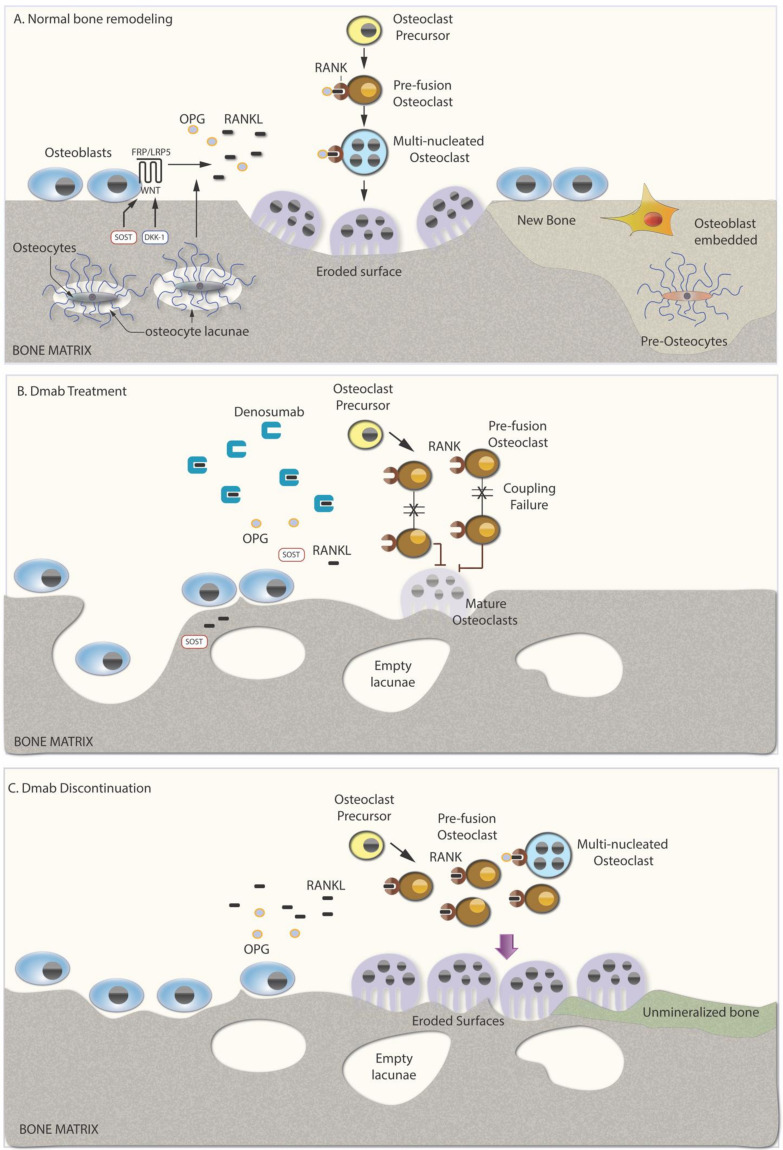
Schematic figure of Denosumab’s mechanism of action. (**A**) Normal bone remodeling. Normally, resorption of old bone matrix is followed by deposition of equal amount of newly formed bone. Osteocytes orchestrate this procedure by producing and secreting sclerostin and Dickkopf −1, and RANKL and OPG, which regulate osteoblastic bone formation and osteoclastic bone resorption, respectively. RANKL binds to its receptor RANK on the surface of osteoclasts and osteoclast precursors, activating these cells. OPG binds to RANKL, preventing it from interacting with RANK. Sclerostin and Dickkopf act as inhibitors of the Wnt intracellular signaling by binding to the frizzled /LRP 5 co-receptor in osteoblasts. (**B**) Treatment with denosumab. Denosumab binds the cytokine RANKL, preventing it from binding to its receptor RANK, and thus it prevents maturation of osteoclast precursors while it promotes apoptosis of mature, multinucleated osteoclasts. Osteoclast number and activity is declined along with bone formation due to the coupling effect. The number of empty lacunae is increased due to accumulation of dead osteocytes that are not replaced by newly embedded osteoblasts/preosteocytes during RANKL inhibition. (**C**) Denosumab discontinuation. Denosumab discontinuation abrogates the suppression on the cells of osteoclastic lineage, leading to increased osteoclastogenesis and subsequently increased osteoblastogenesis. Unmineralized bone is increased due to high bone remodeling rate while the number of osteopetrotic empty lacunae remain unchanged. RANK, receptor activator of nuclear factor kappa-Β; RANKL, receptor activator of nuclear factor kappa-Β ligand; OPG, osteoprotegerin; SOST, sclerostin; DKK-1, Dickkopf; FRP, frizzled related protein; LRP5, low-density lipoprotein receptor-related protein 5.

Under normal conditions of bone remodeling, old and damaged bone is removed by osteoclasts and replaced by newly formed bone matrix laid by osteoblasts in a fine-tuned mechanism [5]. The whole process is tightly regulated by the matrix-embedded osteocytes, which through their extensive lacuna-canalicular network enable the exchange of cellular molecular signals between the bone matrix and the cells lay in the surface [6] (Figure 1a). Within each basic multicellular unit (BMU), osteocytes, as the key players of bone remodeling [7], regulate bone formation through the expression of the Wnt inhibitors sclerostin (SOST) and Dickkopf-1 (DKK-1) [8,9], and bone resorption through the expression of receptor activator of nuclear factor kappa-B ligand (RANKL) [10,11] and its decoy receptor osteoprotegerin (OPG) [12]. In conditions of unbalanced bone remodeling, bone resorption exceeds bone formation, leading to bone loss and structural damage. Increased expression of RANKL by osteocytes, and to a lesser degree by osteoblasts, has a leading role in this negative bone balance in the majority of clinical conditions that induce bone loss. Estrogen withdrawal [13] during menopause or treatment with aromatase inhibitors (AIs), rheumatoid arthritis [14,15], and glucocorticoids [16] are common and characteristic conditions that can lead to an increase in RANKL secretion by osteocytes and osteoblasts, enhancing activation of osteoclast precursors and mature osteoclasts and thus inducing bone loss.

Dmab binds RANKL, thus preventing its binding to RANK on the surface of cells of the osteoclastic lineage. Consequently, Dmab suppresses osteoclast recruitment, maturation, function, and survival, and significantly decreases bone resorption and subsequent bone loss [17,18] (Figure 1b). As a bone antiresorptive agent, its effect on osteoblasts is largely indirect through coupling of resorption and formation within the BMU. Bone histomorphometry analyses in Dmab-treated osteoporotic patients have demonstrated a reduction in both bone resorption and bone formation indices [16]. Recently, it has been shown that the osteocyte lacuna-canalicular network is also affected during treatment with Dmab contributing to differences in bone quality and resistance [19]. Specifically, a reduction in viable osteocytes accompanied by higher numbers of micropetrotic osteocyte lacunae was reported, which is attributed to the retention of old bone due to low bone turnover [19]. In the absence of a direct effect of Dmab on osteocytes, it appears that dead osteocytes accumulate and are not replaced by newly embedded osteoblasts/preosteocytes during RANKL inhibition.

As a monoclonal antibody, Dmab circulates in the bloodstream, binds to secreted RANKL in the extracellular fluid and is cleared from the circulation through the reticuloendothelial system, with a half-life of approximately 26 days. In contrast with bisphosphonates (BPs), Dmab is not incorporated in the bone matrix, and its discontinuation induces significant and abrupt changes in bone remodeling. During the robust inhibition of RANKL, immature preosteoclasts that are unable to resorb bone accumulate in the bone tissue leading to a mass increase in osteoclastogenesis and RANKL release after stopping Dmab (rebound phenomenon) [20,21] (Figure 1c). Histomorphometric analyses of patients who discontinued Dmab without subsequent medication demonstrated increased osteoclast number, osteoclast surface, and eroded bone surface, together with increased osteoblast numbers and osteoblast-covered bone surface [19,22]. On the other hand, the number of empty osteocyte lacunae without the presence of viable cells remained high during discontinuation both in the trabecular and the cortical bone [19] (Figure 1c). Overall, at the tissue level bone structure is compromised after Dmab discontinuation, demonstrating decreased cortical thickness and decreased trabecular bone volume, along with increased amount of unmineralized bone due to rapid acceleration of bone turnover (Figure 1c).

*Summary:* The discontinuation of Dmab leads to significant and abrupt changes in bone remodeling. Enhanced osteoclastogenesis and osteoblastogenesis is evident at tissue level leading to seriously compromised bone structure.

## 4. Discontinuation Effect on Bone Turnover Markers

Dmab discontinuation leads to a rapid, profound increase in the concentrations of BTM, frequently to above pre-treatment baseline levels [4,23]. Even though this phenomenon was initially reported in women with postmenopausal osteoporosis [4,23], it has recently been confirmed for other clinical conditions, such as patients with rheumatoid arthritis receiving glucocorticoids [24,25], and women with breast cancer treated with AIs [24,26].

In postmenopausal women participating in phase 2 and phase 3 trials, investigators described a fluctuating trend for serum CTX that began rising within a mean of 3 months after Dmab discontinuation (9 months following the last injection), with a peak after a mean of 6 months, and returned to pre-treatment concentrations after a mean of 24 months. A similar pattern was found for P1NP, suggesting that remodeling remained coupled during the discontinuation phase [4,27,28].

The pathophysiology of the “rebound effect” on BTM has not yet been fully elucidated. Dysregulation of the Wnt inhibitors SOST and DKK-1 and/or an abrupt increase in expression of RANKL following the loss of effect of Dmab, and/or an increased pool of dormant osteoclast precursors during the treatment period have been proposed [20,21]. In a recent prospective study, levels of DKK-1 and SOST decreased while RANKL concentrations increased 12 months after Dmab discontinuation [29]. However, these changes were not observed in the first months after the Dmab effect had been depleted, leading the authors to conclude that their findings might represent a feedback response to the increased bone turnover (as shown by BTM) and do not support the “dysregulation of Wnt inhibitors” hypothesis. Although the pattern of increase in serum RANKL levels does not fully justify such a phenomenon, a sudden loss of inhibition of the resting osteoclast line after Dmab clearance, with a hyperactivation of these cells was proposed by the authors [29]. In a case-control study, patients with rebound-associated vertebral fractures (RAVFs) following cessation of Dmab treatment had higher serum P1NP and CTX, and lower SOST levels compared with treatment-naïve women with recent osteoporotic vertebral fractures [30]. Furthermore, lower serum concentrations of microRNAs that downregulate osteoclastogenesis and osteoclast activity (miR-503 and miR-222-2), and higher levels of mRNAs of genes involved in osteoclast formation and function (RANK and cathepsin-K mRNA) were found [30].

Weak evidence supports the notion that pre-treatment with BPs might have a protective role after the cessation of denosumab [24,31]. In a small retrospective study, among patients who discontinued Dmab, those pre-treated with BPs showed a smaller increase of CTX concentrations compared to those not previously treated with BPs [31].

The behavior of BTM following Dmab discontinuation probably has a role in the evaluation of the effectiveness of the follow-up antiresorptive treatment.

*Summary:* The discontinuation of Dmab leads to a rapid increase of BTM concentrations, frequently above pre-treatment baseline levels. The pathophysiology of the “rebound effect” on BTMs still remains uncertain. Previous treatment with BPs may have a protective role.

## 5. Discontinuation Effect on Bone Mineral Density

Discontinuation of Dmab is typically associated with a decline in BMD at all skeletal sites. In postmenopausal women treated with Dmab for 24 months and subsequently followed for another 24 months off-treatment, BMD loss at all skeletal sites was evident 6 months after the last injection. The greatest BMD loss in the lumbar spine (LS) was noted at a mean of 18 months off-treatment while both total hip (TH) and 1/3 radius BMD continued to decline up to a mean of 30 months after the last injection [4]. Marked decreases in LS and TH BMD have also been reported in de novo kidney transplant recipients who discontinued Dmab after just one year of treatment [32].

Reports from single centers who monitored their patients following the completion of the pivotal denosumab trials (FREEDOM and its Extension) concluded that the rate and amount of bone loss might be predicted by the total duration of Dmab use: patients treated for a longer period had more pronounced BMD loss at all skeletal sites [23,33]. The rate of BMD loss observed in patients who had stopped Dmab therapy and did not receive any subsequent osteoporosis medication was about 5–11% at all sites during the first year off-treatment [23,27,28,33].

The magnitude of BMD loss after Dmab discontinuation seems to be linked with the phenomenon of multiple vertebral fractures. Indeed, in a post hoc analysis of 1001 participants from the FREEDOM and FREEDOM Extension trials, the patients who sustained multiple vertebral fractures (VFs) following Dmab discontinuation had significantly greater annual BMD loss than those without VFs [34].

To date, it is still uncertain if pre-treatment with BPs preserves BMD gain after Dmab discontinuation. Some evidence suggests that in postmenopausal women, prior BP therapy might not affect the decline of BMD following Dmab discontinuation [35]. In contrast, a recent study reported that patients treated with zoledronate before Dmab initiation had diminished BMD loss compared with initially treatment-naïve patients [36]. Of note, previous BP treatment resulted in smaller BMD increases in patients transitioning to Dmab as compared to treatment-naive patients initiating Dmab [37].

Up to now, no consistent data are available regarding BMD changes after discontinuation in clinical conditions other than postmenopausal osteoporosis.

*Summary:* The discontinuation of Dmab is most commonly associated with a significant decline in BMD at all skeletal sites which tends to return to pretreatment values after 1–2 years off-treatment. The rate and amount of bone loss might be predicted by the duration of denosumab use. The magnitude of BMD loss after Dmab discontinuation seems to be linked to the occurrence of vertebral fractures. It is still uncertain if pre-treatment with BPs prevents BMD loss after Dmab discontinuation.

## 6. Discontinuation Effect on Fracture Risk

Discontinuation of Dmab is associated with a 3- to 5-fold higher risk for vertebral, major osteoporotic, and hip fractures [38,39]. This might be simply a relapse of a given unopposed fracture risk as in the placebo-controlled trials, the off-treatment fracture risk among patients who had received Dmab was not different than that of the placebo group [40]. However, the multiple vertebral fractures are specifically and significantly increased amongst those discontinuing Dmab [34]. The fractures in this setting are typically clinical, occurring a few months after the effect of the last Dmab injection has been depleted [24], and are often described as rebound associated vertebral fractures (RAVFs). Starting from 2016, several case reports or case-series described patients with RAVFs (Table 1).

A post hoc analysis of FREEDOM and FREEDOM Extension did not identify an increased risk of VFs compared to placebo. Namely, among 1001 women who discontinued denosumab after 7 or 10 years of treatment, the annualized risk for VFs rose to 7.1%, similar to the risk in untreated participants. However, among the participants who had sustained at least one VF, the percentage of multiple ones was larger among those who discontinued denosumab (60.7%) than placebo (38.7%; *p* = 0.049), corresponding to a 3.4% and 2.2% risk of multiple vertebral fractures, respectively [34,40]. Of note, in this post hoc analysis, the annualized risk might be underestimated by the relatively short follow-up period (mean 0.2–0.5 years). Another cause of underestimation may be the fact that most studies reporting VFs after Dmab withdrawal were based on follow-up lateral X-rays to identify new fractures and not on MRI which depicts vertebral deformities with greater sensitivity among patients with RAVFs [43]. Recent reports of cohorts from large registries confirmed the increased risk for VFs in Dmab discontinuers [38,39], while the effect of discontinuation on the risk for fractures at other skeletal sites is not clearly estimated as yet. The incidence of VFs after Dmab discontinuation is estimated around 8–10%, with a relative risk of multiple VFs per 100 patient-years of 14.63 (95% confidence interval (CI) 3.3–65.3) [39].

It is important to point out that many of these patients were at high risk for fractures, namely postmenopausal women with severe osteoporosis and prevalent fractures or treated with glucocorticoids or AIs, or even patients who had to interrupt Dmab treatment because they had developed osteonecrosis of the jaw. In this frail population, stopping treatment for osteoporosis would have naturally led to an increased risk of fractures. Nevertheless, new VFs occurred in a relatively short time after the discontinuation (from 6 to 18 months after the last injection, median 10 months). The latest evidence shows that even a 4-month delay in the injection significantly increases VF risk [38]. The increased fracture risk is mirrored by an increase in BTM and a decrease in BMD as previously reported; however, the exact mechanism leading to fractures is yet unknown. Importantly, RAVFs may occur, at least in some cases, sequentially instead of simultaneously [41], therefore, prompt initiation of sequential treatment is of paramount importance.

Taken together, it seems that fractures most commonly occur at the thoraco-lumbar junction, at the same location as the common osteoporotic VFs. This finding implies that despite the higher number and severity of fractures after Dmab discontinuation compared to the commonly seen in insufficiency fractures, the etiopathogenesis may not differ between the two conditions [24,51]. Although the vast majority of cases reported to sustain RAVFs were female, an anecdotal report in men was also recently described [48].

It is not clear whether Dmab discontinuation increases the risk for non-VFs. The detrimental effect of stopping Dmab seems to affect mainly the trabecular bone, probably due to its fast remodeling rate compared to that of cortical bone [24].

*Summary:* The discontinuation of Dmab is associated with an increased risk of multiple, clinical VFs that may occur a few months after the Dmab effect depletion. The risk of non-VFs has not been clearly estimated yet.

### Main Limitations of the Studies

All of the above reported findings are strongly affected by several study limitations such as a weak study design, small sample size, and enrolments focused mainly on women, and in particular elderly patients (mean age >65 years); furthermore, most of the studies did not rule out other comorbidities that might influence bone health. Other confounding factors might be represented by the relatively short off-treatment observation period and other concomitant medication during the off-treatment period [4,22,40]. Finally, another limitation probably is the underreporting of the phenomenon.

## 7. Factors Predisposing to Bone Loss and Fractures Following Discontinuation

According to currently existing evidence, prevalent VF(s) before or during the treatment period are the strongest predictor of new VFs upon discontinuation [24,34]. This association probably suggests that such patients already have compromised bone strength and, therefore, are susceptible to new fractures [24]. Of note, in such patients, Dmab treatment should not be discontinued in the first place, even if their BMD has been improved significantly, as the presence of fracture(s) is indicative of severe bone disease and outweighs the improvement of BMD [59,60]. The rate of BMD loss off-treatment per se could be a risk factor, as it was higher in patients who suffered multiple VFs compared to those with a single VF or without VFs, and higher in case of a single VF compared to no VF [61].

Other factors associated with increased risk of RAVFs, that were identified at the post-hoc analysis of FREEDOM and its Extension follow-up study, include longer duration of the off-treatment period, greater gain in hip BMD with Dmab treatment, and greater loss of hip BMD after discontinuation [34].

The duration of Dmab treatment has been proposed as a factor predisposing to VFs, probably because the higher the number of Dmab doses administered is, the more prominent is the rebound effect [62]. Longer Dmab treatment has been associated with a higher number of VFs [24] and with earlier development of VFs [26]. A finding that indirectly supports this notion is the larger BMD loss after Dmab discontinuation in patients receiving longer Dmab administration [33]. However, the duration of Dmab treatment did not predict multiple VFs in the post-hoc analysis of FREEDOM and its Extension [34].

Vertebroplasty has also been identified as another factor setting patients who discontinue Dmab at risk for VFs, especially at the adjacent vertebrae [24,44,52]. It is possible that bone material properties are compromised even in intact vertebrae, rendering them prone to fracture when increased compressing forces are exerted upon them by the neighboring cemented vertebrae.

Although younger age has been reported to be a risk factor for bone loss after discontinuation [63], RAVFs incidents have been described in a wide range of ages, suggesting that patient’s age is probably of minimal importance [24].

Weak evidence suggests that concomitant AI administration in breast cancer patients may aggravate the withdrawal effect of Dmab on the skeleton, even in normal BMD values, predisposing to VFs [24,26]. Of note, most of these patients do not suffer from osteoporosis before starting AIs and they are usually planned to stop Dmab along with the AI therapy, a strategy that leads them to the rebound phenomenon.

Although previous BP treatment lessens the rebound of BTM, it is uncertain whether it prevents RAVFs. Alendronate failed to avert spontaneous clinical VFs in two high-risk postmenopausal women previously exposed to BPs who discontinued Dmab [47].

*Summary:* Patients with prevalent VFs, greater gain in hip BMD while on Dmab treatment, greater loss of hip BMD after discontinuation, and longer duration of Dmab treatment and of the off-treatment period, are more prone to RAVFs following Dmab discontinuation.

## 8. Patient Management Following Discontinuation

The timing of Dmab discontinuation is a crucial clinical question in the chronic management of an osteoporotic patient. Some experts, based on a post-hoc analysis, suggest that the incidence of non-VFs under Dmab treatment is inversely related with total hip T-score, and the fracture rate decrease reaches a plateau at a T-score range between −2.0 and −1.5, independently of age and prevalent fractures; nevertheless, this is not the case for the vertebral fracture risk which seems to exhibit the same inverse relationship with total hip BMD values but without a restrictive T-score threshold [64]. Therefore, a “treat-to-target” therapeutic approach [65] might be feasible with Dmab therapy, as, in the absence of a high fracture risk profile, BMD monitoring might determine a time point when the risk, at least for non-VFs, has reached a minimal level and no further benefits are expected. However, this approach requires validation in robust prospective scientific studies.

In case Dmab discontinuation is decided, it is the rebound phenomenon in bone remodeling that needs to be quickly counteracted in order to avoid bone loss and to minimize the risk of subsequent multiple VFs [61], although other mechanisms might also be involved [66]. In this context the administration of an anabolic agent such as teriparatide is expected to further increase bone remodeling and thus transiently enhance bone loss especially at cortical sites [67]. Although data are currently lacking regarding the subsequent fracture risk if monotherapy with teriparatide is given following Dmab discontinuation, this option should rather be discouraged at present [61]. Cyclic 6-monthy alternation between teriparatide and Dmab for 3 years preserved BMD at highly cortical sites and total body bone mineral during the 6-month teriparatide administration intervals [68]. However, whether this regimen could be applied in the setting of Dmab discontinuation needs verification in a specifically designed prospective study.

The administration of a subsequent antiresorptive agent following Dmab discontinuation is currently a recommended practice, irrespective of the attained BMD at the time of the transition between treatments. Currently published randomized clinical trials (RCTs) and prospective studies investigating the effectiveness of various antiresorptive agents following Dmab discontinuation are summarized in Table 2. In this context, selective estrogen receptor modulators (SERMs) are an option and are being currently tested in two RCTs (NCT03755193, NCT03623633). However, available data from small case series and case reports suggest that SERMs are not able to prevent bone loss, and probably multiple VFs, in this setting [69,50].

Oral BPs have also been tested regarding their efficacy to prevent bone loss following Dmab discontinuation in studies of different duration and designs. In a study designed to test the adherence, preference, and satisfaction (DAPS study) of patients receiving either Dmab or alendronate, 115 out of the total 250 postmenopausal women with BMD T-scores between −4 and −2 were randomized to receive one year Dmab followed by one year oral alendronate [70,71]. Apart from the lack of rebound of BTM, transition to alendronate maintained or even increased the BMD attained after a single year of Dmab treatment in most of the patients, while BMD decreased in up to 1/5 of patients [71]. Although this seems like a promising result, it corresponds to a very short, and relatively unusual in common clinical practice, period of Dmab treatment. Several small case series have also tested the efficacy of other oral BPs such as risedronate or ibandronate. Specifically, in a case series of 5 postmenopausal women, who received risedronate 35 mg/week for 1 year following Dmab discontinuation, half of the BMD gains were lost [72]; additionally, 5 women on oral BPs following cessation of Dmab in a phase 2 study exhibited smaller BMD decreases compared with those who received no further treatment [28]. A quite short course (3 months) of risedronate failed to prevent bone loss in an observational study of 18 women, as well [73]. This was also the case in a recent observational study published in an abstract form which reported the results of 33 women who switched to BPs following Dmab discontinuation (2 risedronate, 7 ibandronate, and 24 zoledronate); LS BMD decreased significantly compared to that before the transition, while TH BMD was preserved [74].

The two currently published RCTs with zoledronate suggest that the duration of previous Dmab treatment probably has a significant impact on the efficacy of the subsequent antiresorptive treatment to maintain bone gains. Specifically, among postmenopausal women previously treated with Dmab for an approximate period of 2.5 years, a single i.v. administration of zoledronate 6 months after the last Dmab injection preserved the BMD gains for three years in around 80% of the participants [81,46]. However, this was not the case among both female and male patients with an approximate 4.5 years of Dmab treatment, in whom zoledronate did not fully prevent bone loss irrespective of the timing of administration [63]. In a recent observational study in postmenopausal women treated with Dmab for a mean period of 3 years (range 2–5 years), a single zoledronate infusion resulted in retention of 66% and 49% of the LS and TH BMD gains, respectively, after a median period of 2.5 years [78]. In the same study, there was no difference in BMD loss between patients with BMD gains of >9% vs. < 9%, while previous antiresorptive treatment or prevalent fractures had no impact on BMD loss, and all bone loss occurred within the first 18 months after zoledronate infusion [78].

The timing of the subsequent antiresorptive treatment initiation has also been a subject of controversy. It has been speculated that suppressed bone remodeling from the last Dmab injection may reduce the ability of a BP to sufficiently bind to bone surfaces, since the active resorptive sites are limited, thus reducing its efficacy [77]. Although this hypothesis sounds appealing from a pathophysiological point of view, current data suggest that delaying subsequent BP administration at a time point later than 6 months after the last Dmab injection does not add to the performance of the BP [63,82], while it entails the risk of significant bone loss and rebound-associated fractures. Therefore, subsequent treatment should be initiated around the time the effect of the last Dmab injection has been depleted e.g., at 6 months.

Given all the available information and recent recommendations, a rational approach would be the initiation of either oral BPs or zoledronate following Dmab discontinuation. As oral BPs might not adequately consolidate the BMD gains, the measurement of BTM is suggested after 3 months in order to monitor the efficacy and adherence through a level below the mean of healthy premenopausal women (CTX < 280 ng/L, P1NP < 35 μg/L) [61]. BTM measurement could also be useful 6 months after zoledronate infusion, and a second infusion might be considered if a level above the mean of the age and gender-matched controls is found [61]. In any case, treatment should last at least one year while the subsequent annual BMD could guide the decision for the continuation of BPs or not.

*Summary:* Subsequent antiresorptive treatment following Dmab discontinuation is currently a recommended practice to consolidate BMD gains and to avoid the rebound-induced fractures. An at least one-year treatment with either a potent oral BP or i.v. zoledronate is recommended to follow 6 months after the last Dmab injection.

## 9. Conclusions

Dmab is a very effective antiresorptive agent and a useful tool in the long-term management of osteoporosis. However, its favorable skeletal effects can reverse quickly following its cessation [4] due to a rebound increase of osteoclastogenesis [30], which results in a subsequent profound increase of bone turnover, most commonly above pre-treatment values [4], a phenomenon described as “rebound phenomenon”. More importantly, this burst of bone resorption leads to rapid, profound bone loss in most and to increased risk for RAVFs in some of the patients [24,34]. Therefore, subsequent antiresorptive treatment is mandatory, although optimal regimen is yet to be clarified. Current data suggest initiation of zoledronate or alendronate at 6 months following the last Dmab injection; alendronate should be administered for at least one year while in case of zoledronate close monitoring of BTM is suggested in order to (re)administer the agent if BTM remain high [61]. Overall, the duration of subsequent BP treatment is suggested to be 1–2 years. The effectiveness of the subsequent BP treatment to prevent bone loss possibly depends on the total duration of Dmab treatment [61]. Patients with prevalent VFs are more prone to bone loss and RAVFs following Dmab discontinuation.

## 10. Recommendations to Clinicians

- Preventing bone loss upon Dmab discontinuation is an issue of concern.
- There are currently limited data and evidence regarding the optimal management of patients discontinuing Dmab. However, this is a matter of ongoing clinical research and new data are continuously emerging.
- BTM should be measured at 3 months after initiation of an oral BP to monitor adherence and efficacy; the maintenance of BTM below the mean of healthy premenopausal women could be considered as an adequate response [61]. In case of zoledronate infusion, BTM measurement should be performed at 3 and 6 months and if values are increased a repeat zoledronate infusion should be considered. Preferable BTM are serum CTX (its concentration should be maintained below 280 ng/L) and PINP (its concentration should be maintained below 35 μg/L) [61].
- BMD testing should be performed at Dmab discontinuation and at 12 months of subsequent antiresorptive treatment [61,83]. A BMD reduction greater than the least significant change (LSC) should be considered an inadequate response both in case of oral and i.v. BPs and would signify the need of either continuation of oral treatment or of an additional zoledronate infusion. Subsequent BMD monitoring should be individualized, depending on each patient’s clinical condition and therapeutic approach [83].
- Spine X-rays and/or vertebral fracture assessment (VFA) should accompany each DXA measurement to identify new VFs.
- According to current evidence, zoledronate infusion or oral alendronate could be preferred as subsequent treatment in patients discontinuing Dmab. No robust data are available for the use of teriparatide.
- The duration of subsequent BP treatment is proposed to last 1–2 years, although this has not been proven in prospective studies.
- The duration of subsequent BP treatment to prevent bone loss may be affected by the duration of Dmab treatment. Physicians should have this in mind when they plan their treatment strategy.

## Figures and Tables

**Table 1:** Studies reporting rebound-associated fractures after denosumab withdrawal.

Author, Journal, Ref.	Year	Type of Study	*n*. of Patients	Population	Previous Bone Active Treatment	Time from Last Injection to VF	Treatment Duration with Dmab	Number of Fractures/Risk of Fractures	VF before Treatment	Other Bone Active Treatment After	Cause of Discontinuation
Houchen Lyu, Annals of Internal Medicine [38]	2020	Observational	2594	Mean age: 76 y (SD, 10). 94% female, 53% had a history of major osteoporotic fracture.	NA	delay by more than 16 weeks	NA	HR 3.91 (CI, 1.62 to 9.45).	15% of population	NA	NA
Anastasilakis, Bone Rep. [41]	2020	case series	3	**Case 1**: 71 y woman naïve to treatment; **Case 2**: 76 y woman treated with ibandronate for 5 y and treated with GCs; **Case 3**: 53 y male treated with GCs and alendronate for 3 y.	see population	**Case 1**: 8 months and 10 months; **Case 2**: 8 months and 17 months; **Case 3**: 3 months and 2 months of delay in the injection	**Case 1**: 8 y; **Case 2**: 2 y; **Case 3**: 3 y	**Case 1**: 2; **Case 2**: 5; **Case 3**: 5	no	**Case 1**: no; **Case 2**: no; **Case 3**: Dmab	**Case 1**: dentist advice; **Case 2**: NA; **Case 3**: patient discontinued
Kashii, Bone reports [42]	2020	case report	1	60 y woman with osteoporosis complicated by 2 VFs (T6 and T8).	none	12 months	5 doses	5 (T12, L2, L3, L4, and L5)	2 (T6 and T8)	romosozumab 210 monthly, 9 months after last Dmab (fractures occurred after 3 doses of romosozumab	patient negligence
Anastasilakis, Endocrine [43]	2020	Case reports	2	**Case 1**: 66 y woman previously treated with alendronate for 1 y; **Case 2**: 52 y postmenopausal woman with rheumatoid arthritis treated with GCs and methotrexate.	**Case 1**: alendronate; **Case 2**: no	**Case 1**: 9 months; **Case 2**: 9 months	**Case 1**: 10 doses; **Case 2**: 9 doses	**Case 1**: T10, T11, L3, L4; **Case 2**: L1	**Case 1**: no; **Case 2**: no	**Case 1**: na; **Case 2**: Dmab	**Case 1**: physician decision (became osteopenic); **Case 2**: patient negligence
Tripto-Shkolnik, Bone [39]	2020	retrospective	1500	Subjects at least 1 y of treatment. 92% females, mean age = 71.8 ± 9.5 y. Only clinical fractures.	NA	3 months or more	>2 doses	Multiple VFs occurred in 12 (0.8%). The overall rate of fractures per 100 patient-years of follow-up was RR 3.2, (2.2–4.89) the rate of VF RR 4.7, (2.3–9.6) and multiple VF RR 14.6, (3.3–65.3, effect size 1.06).	17%	NA	NA
Gonzalez-Rodriguez E.Breast Cancer Research and Treatment [26]	2019	case series	15	15 women with early-stage breast cancer treated with AI and denosumab 62.3 ± 7.0 years.	NA	7 to 16 months after last denosumab injection (mean 10.9 ± 2.0)	8.2 ± 2.0 doses	from 1 to 11	only 1 patient	Dmab or BPS	10: end of the AI treatment; 1 osteopenia; 2 delayed for dental treatments; 1 omitted; and 1 stopped by the patient.
Fernández Fernández, Reumatol Clin. [44]	2020	restrospective	10	10 women with postmenopausal osteoporosis (66 ± 7.7 y).	90% of population (7 oral BPS, 5 strontium ranelate, 2 raloxifene, 1 tibolone and 1 calcitonine)	8–18 months (10.9 ± 3.3months)	3 to 9 doses, (mean 6 ± 1.7)	2–9	4 patients	TPD: 30%, BPs: 20% Dmab: 20%	2 dental work, 1 low risk of fracture, 7 termination of the time set by the prescribing doctor.
Florez H, Seminars in Arthritis and Rheumatism [45]	2019	case series	7	7 patients with postmenopausal osteoporosis (2 GC) median age was 64 y (56–75 y); 4 patients had previous fragility fractures (2 VF).	5 patients:1 zoledronate1 HRT + BPS2 BPS1 HRT	10 months (8–20)	24–53 months (median 38)	5 (2–8)	2	3 Dmab, 1 combined Dmab and TPD, 3 BPS	2 dental indication; 1 BMD improvement; 1 poor adherence;3 treatment omission and/or delay.
Anastasilakis, JBMR [46]	2019	RCT	57	Treatment naïve postmenopausal women treated with Dmab and achieved osteopenia were randomized (1:1) to receive a single infusion of ZOL (n = 27, given 6 months after the last Dmab injection) or to continue Dmab (n = 30) for 1 year. Follow up until 2 y from randomization.	none	18 months (Zol group), 9 months (Dmab group), 12 months (Dmab group)	2.0 ± 0.2 in Dmab group 2.4 ± 0.2 in ZOL group	2 subjects: 1 new VF and 1 worsening of previous VFs; 1 subject 1 new VF and 2 worsening of previous VF	yes	NA	Osteopenia (design of the trial)
Lamy, osteoporosis International [47]	2019	Case reports	2	**Case 1**: 67 y woman treated with risedronate then raloxifene, then Dmab, then alendronate, finally ZOL; **Case 2**: 68 y woman treated with BPS for 3 y then strontium ranelate finally Dmab for 3 y then alendronate.	**Case 1**: risedronate for 4 years, then raloxifene for 6 years; **Case 2**: BPS for 3 years and strontium ranelate for 2.5 years	**Case 1**: Between 7 and 11 months; **Case 2**: 8 months and 15 months	**Case 1**: 7 doses; **Case 2**: 6 doses	**Case 1**: T8, T9, and L1; **Case 2**: T5, T6, T8, T9, T11, L3, then also T7	**Case 1**: no; **Case 2**: 5 (T12, L1, L2, T10 and L4)	**Case 1**: alendronate and ZOL; **Case 2**: alendronate then TPD	**Case 1**: BMD gain and treatment duration; **Case 2**: osteopenic
Anagnostis P, Journal of Clinical Rheumatology [48]	2019	letter to the editor	2	**Case 1**: 45 y male with osteoporosis complicated by multiple vertebral low-energy fractures (T7;T10-L5); **Case 2**: 80y man with osteoporosis complicated by VFs (T9 and L1 to L5) in the context of GCs treatment for polymyalgia rheumatica.	**Case 1**: TPD; **Case 2**: none	**Case 1**: 12 months; **Case 2**: 14 months	**Case 1**: 3 doses; **Case 2**: 2.6 doses	**Case 1**: 3; **Case 2**: 2	**Case 1**: 9; **Case 2**: 6	**Case 1**: Dmab; **Case 2**: ibadronate then Dmab again	**Case 1**: patient omission for musculoskeletal pain; **Case 2**: general practitioner switch to ibandronate because osteopenia
De Sousa SMC, Clin Endocrinology [49]	2019	case report	1	70 y woman with postmenopausal osteoporosis and no prior fractures.	Combined hormone replacement for eight years; risedronate for eight years, including one year of concomitant treatment with raloxifene; strontium for two years.	7 and 8 months	7 doses	1 after 7 month, 1 after 8 months, 2 after 10.5 months and also rib fractures	0	Dmab after 9 months (3 months delay)	dental issue
Gonzalez-Rodriguez, Case Reports in Rheumatology [50]	2018	Case report	1	60 y woman in AI therapy with letrozole.	none	10 months	12 doses	T11 and L5	no	raloxifene then Dmab again after VFs	end of AI and osteopenia
Che H., Osteoporosis International [51]	2018	Restrospective	8	Subject with VF cascade defined as 3 or more VF in 1 year. 8 patients after Dmab, in a pool of 135 patients.	7 BPS	5 subjects: 4–6 months and 3 subjects: 14–18 months	NA	multiple VF	5 patients	NA	NA
Tripto-Shkolnik, Calcified Tissue International [52]	2018	phone survey	5	9 female 74.2 ± 5.3 years.	6 BPS, 1 tpd plus BPS	6.5 ± 4.7 months	4.9 ± 1.6 doses	36 in 9 patients	6 patients	Dmab or zol	4: physician decision; 1: administrative; 3 non-osteoporosis related medical condition; 1 unknown
R. Niimi, Osteoporosis International [53]	2017	case report	1	69 y woman with severe osteoporosis (L1 fracture)	naïve	10 months	5 doses	5	1	Dmab	Maxillitis
Cummings, JBMR [34]	2017	RCT	1471	Among 1001 participants who discontinued treatment during FREEDOM or Extension with >7 months of follow-up after the last dose (1001, denosumab; 470, placebo).	At least 1 year washout from eventually previous treatment.	>7 months	2–19 doses	56 subjects, 36 (61%) multiple VF, 23 subjects non-VF	24% of population	14% received BPS	End of study period
Anastasilakis, Popp, Polyzos, Lamy, Aubry-Rozier), JBMR [54,55,56,57,58]	2016–2017	case series	24	24 postmenopausal women (age 64.1 (48–83))	strontium ranelate and raloxifene; (n1)TPD(n1), and BPS (n2)	11.2 (8–16) months	6 doses (2–10)	4.7 (1–9)	33% of population	5 patients were subjected to vertebroplasty, all unsuccessful. TPD was the most commonly prescribed alone or in combination with Dmab or Dmab alone	13 osteopenic; 1 end of AI; 5 patient’s wish or negligence; 1 dental issue; 3 Dmab treatment duration

Abbreviations: AI: aromatase inhibitors; BPs: bisphosphonates; Dmab: denosumab; GCs: glucocorticoids; HR: hazard ratio; HRT: hormone replacement therapy; NA: not available; RR, relative risk; TPD: teriparatide; VF: vertebral fractures; y: years; ZOL: zoledronate.

**Table 2:** Studies evaluating the efficacy of antiresorptives following denosumab discontinuation *.

Study, Year	Study Type,	*n* (% Female)	Dmab Duration	Post-Dmab Antiresorptive Regimen	% LS BMD Change (% Mean Dmab Gain Preserved) (% pts Preserved BMD **)	% TH BMD Change (% Mean Dmab Gain Preserved) (% pts Preserved BMD **)	% FN BMD Change (% Mean Dmab Gain Preserved) (% pts Preserved BMD **)	VFs	Non-VFs	Comments
Freemantle, 2012 [70]	RCT	115 (100)	1 y	ALN, 1 y	0.6 (100) (NR)	0.4 (100) (NR)	−0.1 (100) (NR)	0	1 humerus	− DAPS study—primary aim: compliance
Lehmann, 2017 [75]	Case series	22 (100)	2.5 y	ZOL, 1 infusion	−3.8 (61.2) (NR)	−1.7 (56.4) (NR)	−0.6 (73,9) (NR)	0	1 calcaneous	− BMD measured 2.5 yrs after ZOL
Leder, 2017 [76]	Follow-up of RCT	28 (100)	2 or 4 y	1 y, ZOL (*n* = 8); ALN (*n* = 8); IBN (*n* = 2); Dmab (*n* = 10)	−1.2 (NR) (NR)	NR (NR) (NR)	−0.6 (NR) (NR)	0	1 tibia (stress)	− DATA follow-up − 36% of pts received Dmab
Reid, 2017 [77]	Case series	6 (100)	7 y	ZOL, 1 infusion	−9.2 (50.3) (NR)	NR (NR) (NR)	NR (NR) (NR)	NR	NR	− Follow-up of FREEDOM pts − BMD reported 2 y after ZOL
Horne, 2018 [72]	Case series	16 (100)	2 y	1 y, ZOL (*n* = 11), RIS (*n* =5)	ZOL: −5 (73) (NR) RIS: −9.9 (41) (NR)	ZOL: −1.5 (87) (NR) RIS: −3.9 (64) (NR)	NR (NR) (NR)	NR	NR	− Follow-up of FRAME pts (1 y romosozumab or placebo before Dmab) − ZOL was given with up to 6 mo delay
Anastasilakis, 2019 [46]	RCT	27 (100)	2.4 y	ZOL, 1 infusion	12 mo: 1.7 (100) (NR) 24 mo: 0.1 (100) (11.1)	NR (NR) (NR)	12 mo: NR (100) (NR) 24 mo: NR (100) (14.8)	1	0	− After Dmab study
Everts-Graber, 2020 [78]	Retrospective observational	120 (100)	2–5 y (mean 3 y)	ZOL, 1 infusion	−3.3 (66)	−2.2 (49)	−1.5 (57)	3	4 (1 calcaneus, low energy—1 distal radius, low energy—1 pubic, high energy—1 humerus, high energy)	− BMD measured 2.5 y after ZOL
Everts-Graber, 2020 [79]	Retrospective	193 (100)	mean 2.5 y	ZOL 1 infusion (*n* = 171), OR Other (*n* = 22) (IBN (*n* = 6), ALN (*n* = 10), SERMs (*n* = 6))	ZOL: −3.6 (NR) (NR) Other: −3.2 (NR) (NR)	ZOL: −2.5 (NR) (NR) Other: −3.4 (NR) (NR)	ZOL: −1.6 (NR) (NR )Other: −3.4 (NR) (NR)	5 (3 on ZOL, 1 on IBN, 1 on SERM)	3 (all on ZOL)	− The 120 pts of the previous study were included to this study − BMD measured 1–4 y (median 26 mo) after ZOL
Kendler, 2020 [71]	Post-hoc analysis of RCT (see DAPS above)	115 (100)	1 y	ALN, 1y	0.6 (100) (84.1)	0.4 (100) (92.4)	−0.1 (100) (78.3)	0	1 humerus	
Kondo, 2020 [80]	Retrospective observational	30 (96.7)	<3 y (average 1.5 y)	ZOL, 1 infusion	1.8 (100) (NR)	NR (NR) (NR)	2.1 (100) (NR)	0	0	
Laroche, 2020 [73]		18 (100)	1–4 y (mean 39 mo)	RIS, 3 mo + 9 mo follow-up without RIS	−4.6 (NR) (NR)	−1.8 (NR) (NR)	NR (NR) (NR)	1	0	
Makras, 2020 [81]	Extension of RCT (see afterDmab above)	23 (100)	2.4 y	ZOL, 1 infusion	36mo: −1.75 (100) (82.6)	NR (NR) (NR)	36 mo: NR (100) (95.6)	0	1 metatarsal	− In 4 pts LS BMD and in 1 pt FN BMD decreased to T-score < −2.5
Solling, 2020 [63]	RCT	59 (88.5)	4.6 y	ZOL, 1 infusion 6 mo after last Dmab dose (6M) OR 9 mo after last Dmab dose (9M) or when turnover increased (OBS)	6M: −4.8 (NR) (65) 9M: −4.1 (NR) (65 )OBS: −4.7 (NR) (63)	6M: −2.6 (NR) (15) 9M: −3.2 (NR) (35) OBS: −3.6 (NR) (37)	6M: −3.0 (NR) (20) 9M: −3.5 (NR) (30) OBS: −4.6 (NR) (37)	2	2 (1 rib, low energy—1 humerus, high energy fracture)	

**Abbreviations:** ALN, alendronate; BMD, bone mineral density; Dmab, denosumab; FN, femoral neck; IBN, ibandronate; LS, lumbar spine; mo, months; *n*, number of patients; NR, not reported; pts, patients; RCT, randomized clinical trial; RIS, risedronate; SERM, selective estrogen receptor modulator; TH, total hip; VF, vertebral fracture; non-VF, non-vertebral fracture; y, year; ZOL, zoledronate. * case reports and small case series (<5 subjects) are not reported in the Table. ** different definitions were used among studies to define when a patient would be considered as having preserved his/her BMD.

## Data Availability

Data sharing not applicable.
